# Emotion perception bias associated with the hijab in Austrian and Turkish participants

**DOI:** 10.1177/17470218211048317

**Published:** 2021-09-29

**Authors:** Sebastian Korb, Tugba Ceren Deniz, Bengi Ünal, Alasdair Clarke, Giorgia Silani

**Affiliations:** 1 Department of Psychology, University of Essex, Colchester, UK; 2 Department of Cognition, Emotion, and Methods in Psychology, University of Vienna, Wien, Austria; 3 Faculty of Arts and Sciences, Department of Psychology, TED University, Ankara, Turkey; 4 Department of Clinical and Health Psychology, University of Vienna, Wien, Austria

**Keywords:** Facial expressions, prejudice, hijab, Islam, cross-cultural

## Abstract

In a cross-cultural study, we investigated the link between explicit attitudes towards the hijab and implicit measures of cultural and religious bias during the recognition of emotions. Participants tested in Austria (*N* = 71) and in Turkey (*N* = 70) reported their attitude towards the hijab, and categorised in a mousetracker task happy and sad faces of women, shown with five levels of intensity, and framed either by a hijab or by an oval-shaped mask. The two samples did not differ in their explicit attitudes towards the hijab. However, negative attitude towards the hijab predicted greater sadness attribution to happy faces with the hijab in Austrian participants. Unrelated to their explicit attitudes, Turkish participants attributed more sadness to happy faces with than without the hijab. Results suggest that the sight of the hijab activated, in both Austrian and Turkish participants, implicit biases resulting in associations with sadness and negative emotions.

## Introduction

In the last decades, many Western countries have witnessed an increase in the number of immigrants and asylum seekers from countries in which the main religion is Islam. This has partly led to tensions and resentment, and in some cases to outright hostilities towards Muslim immigrants (Amnesty International, 2018; Awan & Zempi, 2017 https://tellmamauk.org/category/reports/). Part of the societal struggle related to immigrant integration and acceptance may stem from the misinterpretation of intentions, behaviours, and even emotional expressions, possibly due to negative associations with immigrants’ cultural and religious symbols. For example, Muslim women wearing the hijab (headscarf), and even more so those wearing the niqab (face veil), remain an unfamiliar sight to many Western Europeans and may evoke fear or disapproval in parts of society (Moors & Salih, 2009). Even those who generally favour a multicultural society may have strong prejudices about the headscarf. This religious prejudice against Islam was shown to hinder Muslim’s integration in Western societies (Kunst et al., 2016). But can these prejudices and stereotypes also affect facial emotion recognition?

Past research has indeed suggested that people from a non-Islamic background perceive emotional expressions differently in women wearing Islamic vs. secular garments covering parts of the head/face. For example, Kret and de Gelder (2012) showed to Dutch participants pictures of women wearing the hijab or niqab, and of women wearing a non-religious cap and scarf. More fear and less happiness were found to be attributed to women with Islamic face cover. Importantly, most effects were found exclusively or predominantly in response to pictures of women wearing the niqab, which hides the entire face leaving only the eye region uncovered, and which is a rather rare sight, not only in the Netherlands, but even in most Islamic countries. The more common hijab, on the other hand, did not strongly influence participants’ emotion attribution in the study by Kret and de Gelder (2012).

In a follow-up study (Kret & Fischer, 2018), female faces wearing either a niqab or cap and shawl, and male faces wearing a turban or cap and shawl, were shown for 40 ms with happy, angry, sad, or fearful expressions. Dutch students categorised each picture by picking one of four emotion labels. Emotion recognition, especially of happiness, was found to be more accurate for faces with cap and shawl (participants’ ingroup), than for faces with Islamic head-dress (the outgroup). When emotional miscategorisations occurred, the “sadness” label was chosen more often and more quickly for outgroup (wearing a niqab or turban) compared with ingroup faces.

In line with this, numerous studies have found a cultural ingroup advantage for the recognition of emotional facial expressions (for a review see Elfenbein & Ambady, 2002). Less accurate emotion recognition was also shown for outgroup members who are from our own culture (Thibault et al., 2006), even when group assignment is random and recent (Lazerus et al., 2016). Facial mimicry, believed to contribute to emotion recognition (Wood et al., 2016), is also reduced for outgroup members (van der Schalk et al., 2011; but see Sachisthal et al., 2016). Emotion recognition biases due to stereotypes and prejudices may take place already at the perceptual level, as conceptual knowledge is known to influence visual perception in a top-down manner (Dunning & Balcetis, 2013; Brooks & Freeman, 2018). In agreement with this, the interpretation of the same facial expression can differ dramatically depending on the scene it is embedded in (e.g., body posture), and this process appears to be automatic (Aviezer et al., 2008, 2011). Moreover, Dutch participants perceived anger more rapidly in Moroccan faces, and sadness more rapidly in Dutch faces, and this effect was predicted by their implicit associations between the terms Moroccan-anger, and Dutch-sadness (Bijlstra et al., 2014).

To summarise, past research suggests that our values and beliefs can bias our ability (down to the perceptual level) to recognise emotional facial expressions in outgroup individuals, including people from another culture or religion. Happy women wearing the niqab tend to be miscategorised as sad by Western participants, who probably associate Islamic head covers with negative stereotypes about the Islam (Kret & de Gelder, 2012; Kret & Fischer, 2018). This misattribution of sadness to happy faces with Islamic head covers was found despite the fact that happy faces are typically recognised faster and more accurately, compared with other emotional expressions (a phenomenon often called the “happy face advantage,” e.g., see Calvo & Lundqvist, 2008; Palermo & Coltheart, 2004; Tottenham et al., 2009). Someone’s explicit negative associations with Islam are not always, however, directly related to their emotion recognition bias (Bijlstra et al., 2014; Kret & de Gelder, 2012). It also remains unclear, exactly how much of this bias extends to faces of women wearing the hijab, which is a more moderate and revealing head cover, compared with the niqab or burqa. Moreover, the majority of past research has focused on Western participants, presumably from a non-Islamic background. It is currently unknown, if similar effects can be found in a predominantly Islamic country. This cross-cultural comparison seems relevant not only based on the assumption that the hijab can evoke negative and positive associations in, respectively, Western and Turkish participants. Past research has shown that cultural differences can also affect basic cognitive processing, including object perception (Kitayama et al., 2003).

To address these points, we measured in Austrian and Turkish samples (1) the explicit attitude towards the hijab, and (2) the effects of implicit bias about the hijab during an emotion categorisation task, in which faces of women wearing the hijab (or with a superimposed oval background mask) were shown with five degrees of linearly increasing emotional intensity. We chose to present emotions of happiness and sadness based on the previous finding that less happiness is perceived in faces wearing an Islamic head-dress (Kret & de Gelder, 2012; Kret & Fischer, 2018), and based on the reflection that sadness is encountered more frequently in every-day life, compared with fear. Austria and Turkey were chosen as they differ in terms of the cultural prevalence, and the legal and societal acceptance, of the hijab. Austria is a predominantly Christian country and has a generally low acceptance of Islamic garments in public, as suggested by its ban of the headscarf for girls below the age of 14 (Oltermann, 2019; Schuetze & Bennhold, 2020). Turkey’s dominant religion is Islam, although it is, by constitution, a secular state. Wearing the hijab is culturally accepted in Turkey and has been allowed in recent years in universities, government buildings, schools, and the armed forces (“Turkey,” 2020). People living in Turkey are therefore likely to have a more positive association with the hijab, compared with people living in Austria.

To measure explicit attitude towards the hijab, participants completed a self-report questionnaire, based on Kret and de Gelder (2012). The mousetracker task (www.mousetracker.org) was used to assess if emotion categorisation in a two-alternative forced choice task is influenced by implicit bias, as it allows to continuously track the trajectories of motor responses corresponding to the mouse movement towards (and away from) response options, from which the real-time course of mental processes (and biases) can be inferred (Freeman, 2018; Freeman & Ambady, 2010). Concretely, after starting each trial with a mouse click at bottom of the screen, participants indicated the emotional expression of an appearing face by moving the mouse cursor to click on either the happiness or sadness labels, presented at the top corners of the screen. Faces displayed five increasing levels (20% to 100%) of happiness or sadness. Participants were instructed to respond as fast as possible, and reminded to do so after slow trials. An interesting aspect of the mousetracker task is that it provides, in addition to accuracy and response time (RT), a measure of the degree of deviation of the mouse trajectory towards the incorrect category label. The greater the deviation, measured as area under the curve (AUC), the greater the competition of the correct and incorrect labels, and thus of their underlying representations. The AUC often correlates with, but is not equivalent to RT (Freeman, 2018), and can thus provide an additional measure compared with other tasks like the implicit association test (Greenwald et al., 1998).

The following hypotheses were made based on the literature. H1: explicit attitude towards the hijab will be more negative in Austrian compared with Turkish participants. Participants with a more negative attitude towards the hijab will (H2) categorise happy faces with the hijab more frequently as sad, (H3) have a larger AUC towards the sadness label when correctly recognising happy faces with the hijab, and (H4) have slower RTs when correctly recognising happy faces with the hijab. H5: The effects described in H2–4 will be more pronounced in the Austrian compared with the Turkish sample. H6: The effects described in H2–4 will be more pronounced for low-intensity faces.

## Method

We carried out two separate experiments, one in Austria and one in Turkey, using the same stimuli, task, and procedure—with the exception that the experiment carried out in Turkey included faces with and without a hijab, while only faces wearing a hijab were included in the Austrian experiment. The reason for this difference is that we aimed to maximise statistical power for finding an effect in the Austrian sample, which was tested first, and later improved the design by also including a control condition in the Turkish sample.

### Participants

Sample size was determined before data acquisition. To define the required sample size, we took as a reference the number of participants tested in a published study investigating perception of race and gender in faces with the mousetracker task (Johnson et al., 2012; 77 in Study 1 and 66 in Study 2). Moreover, a power analysis with the software G*Power (Faul et al., 2007), assuming a medium effect size of Cohen’s *d* = 0.5, with alpha set to 0.05 and power to 0.8, suggested a minimum sample size of 128 (64 per cultural group). Slightly more participants were recruited (74 in Austria, 71 in Turkey), to account for eventual data loss. Three subjects from the Austrian subsample were excluded from analyses, because they had given the same response to every question in the questionnaire (one male), suffered from strabismus (one male), or because they wore a hijab, which is uncommon in Austria and was therefore considered an outlier feature.^1^ One participant from the Turkish sample was excluded, as he had chosen the wrong emotion in 78% of trials. Eight participants in the Turkish sample wore the hijab, but were kept in the analyses, as this is common in Turkey. The final sample included 71 participants in Austria and 70 participants in Turkey (see Table 1). Participants were recruited from university participant pools (https://www.sona-systems.com) and through local advertising. The study was approved by the ethics committees of the respective universities in Austria and Turkey (reference numbers respectively 00472 and 2019/66), and all participants gave written consent.

**Table 1.** Demographic characteristics of the Austrian and Turkish samples (unknown religion corresponds to participants’ choice not to indicate their religious belief).

	Austrian sample	Turkish sample
*N*	71	70
*N* females	43	57
Age range	20–35	19–35
Age mean (*SD*)	25.96 (3.98)	22.76 (3.30)
Nationality	Austria (40), Germany (19), Bosnia (1), Colombia (1), Poland (1), Romania (2), Russia (1), Slovakia (1), Ukraine (3)	Turkey (70)
Religion	Christian (35), Atheist (1), Muslim (3), Other (1), Unknown (31)	Muslim (51), Agnostic (1), Deist (1), Unknown (17)

### Stimuli

Pictures of eight different adult female faces with neutral, happy, and sad expressions (all with closed mouth) were taken from the NimStim database^2^ (Tottenham et al., 2009). Although their precise ethnicity is unknown to us, we chose seven faces that look white (Caucasian), and one face that has slightly more Asian traits. Importantly, we were careful not to select faces that would stand out in racial terms (very different ethnic background, e.g., Black Americans) in either country. Using GIMP (version 2.10.10), every face was overlaid with the same photo of a hijab covering hair and neck, or with an oval mask. Four intermediate levels of increasing happiness and sadness were created using the software FantaMorph (www.fantamorph.com), which automatically detects facial features and places key dots on appropriate positions. Fully neutral faces were discarded. The final stimulus set included 160 different faces (examples: https://bit.ly/3leFGtk): eight identities × two emotions (happy, sad) × five intensities (20, 40, 60, 80, 100%) × two contexts (hijab, oval). All faces were sized 479 × 600 pixels and shown with approximately 11 × 14 degrees of visual angle. Only faces with the hijab were shown to the Austrian sample.

### Task

**Figure 1.**
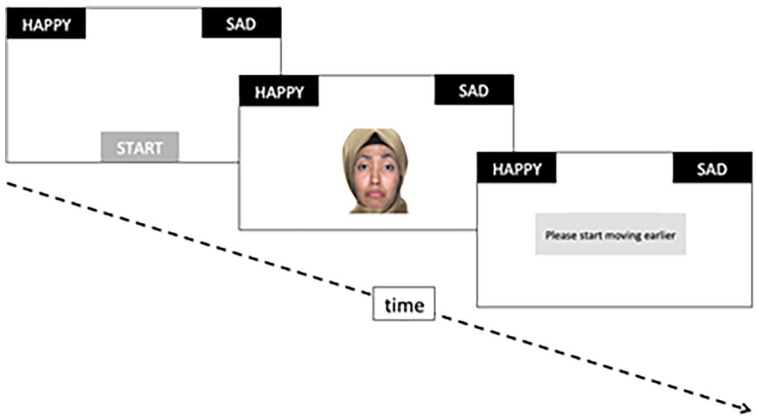
Example trial in the mousetracker task. Actual labels were in German/Turkish, and in smaller font size.

The mousetracker task (www.mousetracker.org; Freeman, 2018; Freeman & Ambady, 2010) was used to measure participants’ bias in the categorization of women’s facial expressions. Participants were instructed to start each trial by clicking with the left mouse button on the word “start” appearing at the centre-bottom of the screen (Figure 1). A face then appeared in the central lower half of the screen, and participants indicated whether they perceived its expression as happiness or sadness, by rapidly moving the mouse cursor up and sideward until clicking on the respective labels, which appeared always on the left and right top corners of the screen. The left/right side of the happiness/sadness labels was counterbalanced across participants. Participants received no feedback about their response, but were invited to respond faster if the onset of mouse movement was later than one second after the face onset.

### Procedure

Data were acquired in university laboratories in Vienna and Ankara. Participants were informed that the purpose of the study was to investigate how the hijab influenced the perception of emotional facial expressions. They were seated circa 50 cm in front of a 21- or 23-inch screen with a resolution of 1920 by 1080 pixels, connected to a PC running on Windows 10, equipped with a standard keyboard and optical mouse. Participants completed 10 practice trials, in which a female identity (not part of the main task) was shown. They then completed three (Austrian sample) or four (Turkish sample) blocks of 80 trials each, with the possibility to pause between blocks. In the Austrian sample, only faces with a hijab were included, and each of the 80 stimuli was shown three times, for a total of 240 trials. The Turkish sample additionally saw the same faces with an oval mask instead of a hijab, and each of the 160 stimuli was repeated once, for a total of 320 trials. The order of stimuli in each block was random and different for each participant. After completing the task, participants filled out an online questionnaire to assess their explicit attitude towards women wearing the hijab (henceforth called “Attitude”). Concretely, they indicated on a 7-point Likert-type scale, from 1 (*Strongly disagree*) to 7 (*Strongly agree*), their levels of acceptance, admiration, affection, antipathy, approval, contempt, disapproval, hostility, and sympathy, and indicated how much they thought that women wearing the hijab were warm (see Supplementary Material).

### Analyses

Data and analysis scripts are available online (https://bit.ly/3leFGtk). For both studies, we report all measures, manipulations, and exclusions. Participants’ attitude towards the hijab was scored by summing the questionnaire items (after reversing coding questions 4, 6, 7, 8, see Supplementary Material).

To analyse percentage of errors, we fitted a generalised linear mixed-effect binomial model (GLMM) with the *glmer* function of the *lme4* package in R (Bates et al., 2014; R Core Team, 2019). Separate linear mixed effects models (LMMs) were fitted, using the *lmer* function from the *lmerTest* package, on the log-transformed reaction times (RTs) and on the area under the curve (AUC), after excluding trials with categorisation error, and those with a RT more than the mean plus two times the standard deviation of all trials.

A first set of analyses was conducted over all participants, excluding trials with the oval mask (only shown to Turkish participants). These models included the fixed effects Emotion (happy, sad), Intensity (20%, 40%, 60%, 80%, 100%), Country (Austria, Turkey), and the continuous predictor Attitude. A second set of analyses was carried out on the data from the Turkish sample alone, to compare responses to faces with and without the hijab. These models included the fixed effects Emotion, Intensity, Condition (Hijab, Mask), and Attitude. Categorical predictors (Emotion, Country, Condition) were centred through effect coding (e.g., −1, 1), continuous predictors (Intensity, Attitude) were mean-centred and scaled. By-subject and by-stimulus random intercepts and random slopes for within-subject predictors (Emotion, Intensity, Cover), as well as their interactions, were included as random effects.^3^ In the Results section, we report significant main or interaction effects with the predictor Attitude, and with the predictor Cover (Turkish sample). Please refer to model tables in the Supplementary Materials to see all statistics. Simple slopes post hoc comparisons were carried out with the *sim_slopes* function of the *interactions* package. Figures were created using the packages *ggplot2* and *cowplot*; model tables with the function *tab_model* from the package *sjPlot*.

## Results

### Comparison Austria vs. Turkey

**Figure 2.**
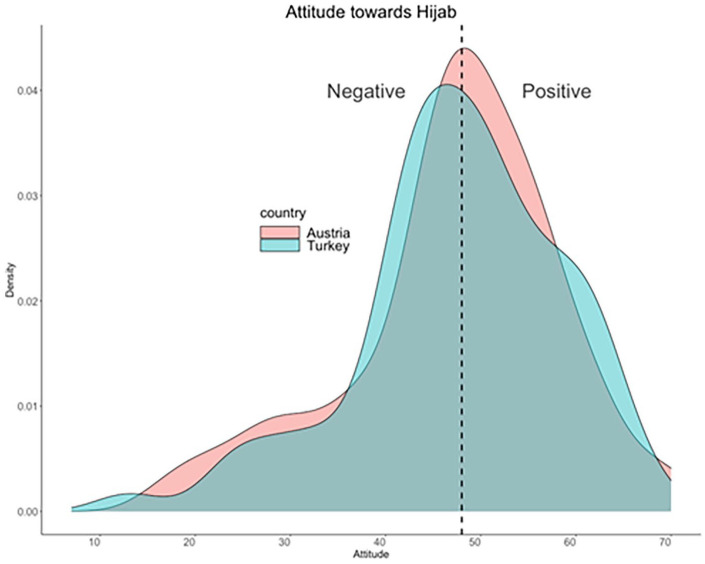
The distribution of attitudes towards the hijab did not differ between the Austrian and the Turkish sample, and the median (48, see dashed vertical line) was identical. A lower score on the x-axis indicates a negative attitude (less acceptance and sympathy, more hostility), and a higher score a positive attitude (more acceptance and sympathy, and less hostility).

Participants’ explicit attitude towards the hijab was, overall, rather positive. The distribution of attitudes was similar, and the median (48) was identical across the Austrian and Turkish samples (Figure 2). The hypothesis of a more negative attitude in the Austrian sample (H1) was not confirmed.

#### Categorisation errors

**Figure 3.**
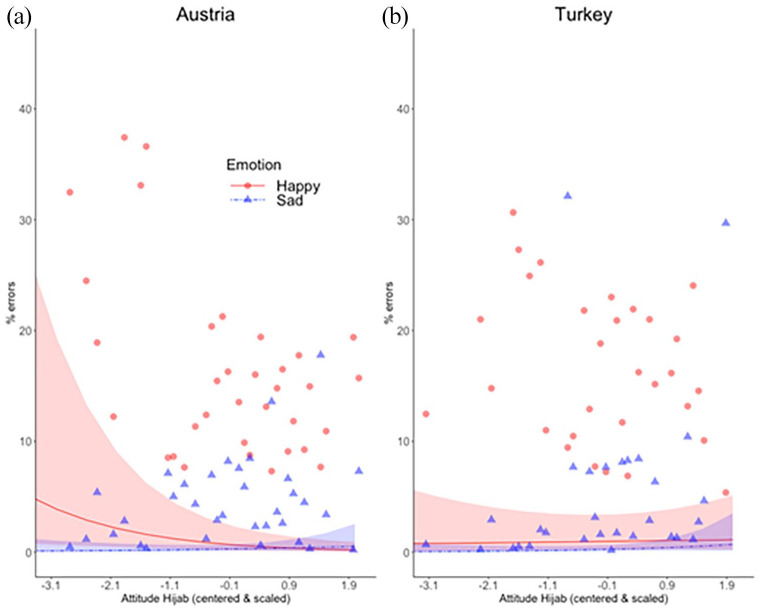
A significant Emotion × Attitude interaction reflected higher percentage of errors for happy than sad faces by participants with a more negative attitude towards the hijab. When splitting by country, this interaction remained significant in the Austrian, but not in the Turkish sample. Lines indicate the fitted distributions, coloured areas the 95% confidence interval. Marginal means, averaged at each level of Attitude, are shown for happy faces (red circles), and sad faces (blue triangles).

The GLMM on categorization errors resulted in a significant Emotion × Attitude interaction (*b* = .33, *z* = 2.12, *p* = .03). In line with H2, a higher percentage of errors was found for happy than sad faces by participants with a more negative attitude towards the hijab (Figure 3).^4^

To further probe for intercultural differences (H5), we split the data and fitted the same GLMM (minus the predictor Country) to the Austrian and Turkish samples separately. The Emotion × Attitude interaction was significant in the Austrian (*b* = .44, *z* = 2.12, *p* = .03) but not in the Turkish sample (*b* = .23, *z* = 2.12, *p* = .3). The same pattern of results was found after randomly excluding one third of trials from the Austrian sample (selecting them by subject, emotion, and intensity), to obtain the same number of trials with hijab for both countries (see model table in Supplementary Material). These results, which should be considered preliminary evidence due to the lack of a significant three-way interaction with Country, support H5, i.e., that the Emotion × Attitude interaction is more pronounced in the Austrian sample.

#### Area under the curve (AUC)

**Figure 4.**
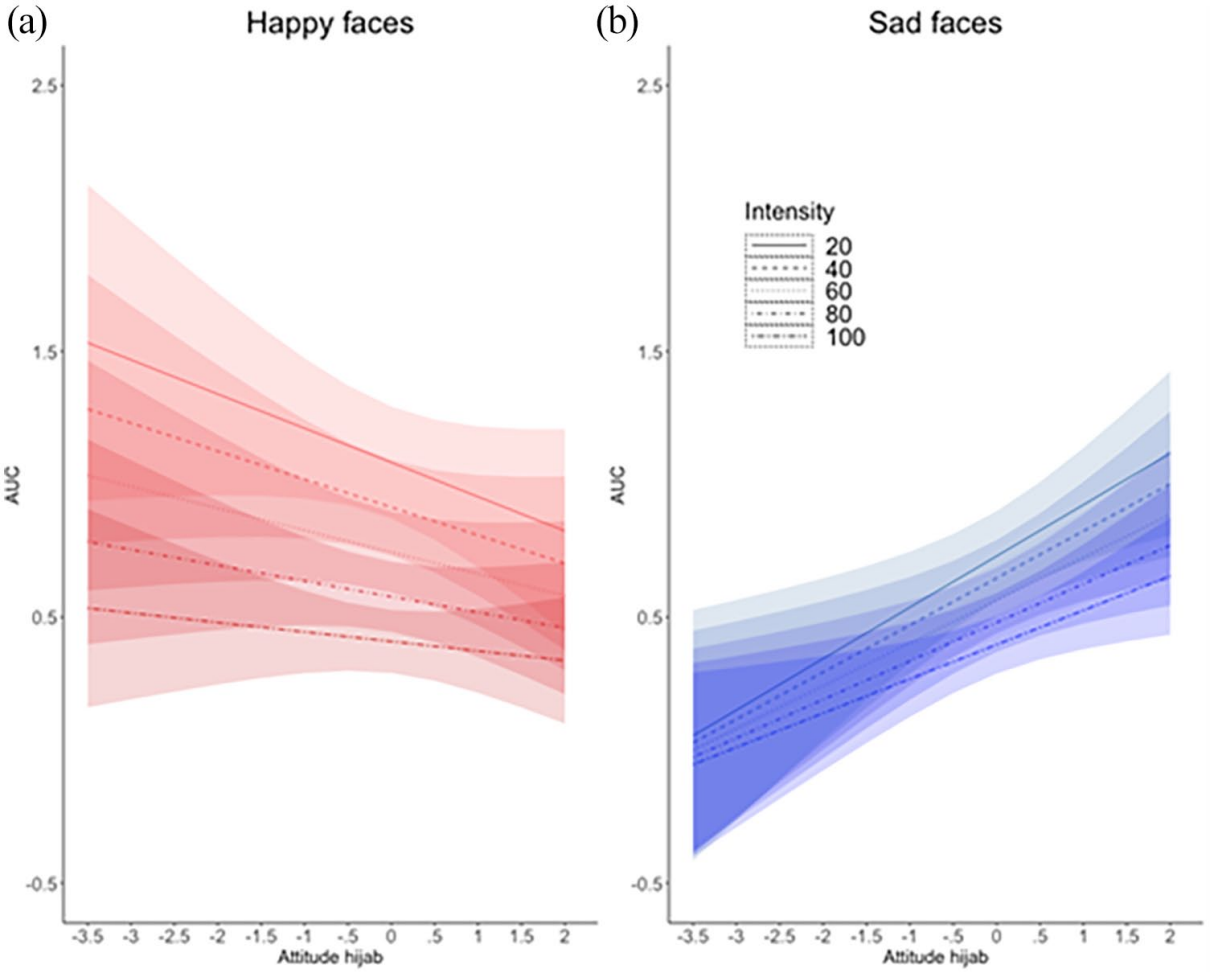
The AUC in correct trials was larger for (a) happy compared with (b) sad faces, as indicated by a significant Emotion × Attitude interaction, and this effect was larger for low-intensity expressions, as suggested by an Emotion × Intensity × Attitude interaction that fell just short of significance (*p* = .055). Analyses separately by country showed that both interactions were significant in the Austrian but not in the Turkish sample. Lines indicate model fits, coloured areas the 95% confidence interval.

The LMM on AUC of correct responses resulted in a significant Emotion × Attitude interaction, *b* = .24, *t*(131.82) = 3.23, *p* = .002, and in an Emotion × Intensity × Attitude interaction, which fell just short of significance, *b* = −.05, *t*(115.67) = −1.93, *p* = .055. Simple slopes analyses of the Emotion × Attitude interaction revealed that, for an increasingly positive attitude towards the hijab, the AUC for happy faces decreased significantly (*b* = −.09, *p* = .03), and the AUC for sad faces increased significantly (*b* = .10, *p* = .01). H3 was thus confirmed. Moreover, and in line with H6, this pattern was more pronounced for low intensities of emotional expression, as revealed by simple slopes analyses of the Emotion × Intensity × Attitude interaction (Figure 4).

Explorative analyses fitting the same model to the Austrian and Turkish samples separately showed that the Emotion × Attitude, *b* = .12, *t*(69.15) = 3.53, *p* < .001, and the Emotion × Intensity × Attitude interaction, *b* = −.03, *t*(14620) = −3.07, *p* = .002, were significant in the Austrian, but not in the Turkish sample (both *p* > .1). The same pattern of results was found after randomly excluding one third of trials from the Austrian sample. These results speak for H5, i.e., that the effects on AUC are more pronounced in the Austrian compared with the Turkish sample.

#### Reaction time (RT)

The LMM on RT of correct responses resulted in a significant Emotion × Attitude interaction, *b* = .02, *t*(125.1) = 2.08, *p* = .04, which supports H4. However, neither the slope for happy faces (*b* = .01, *p* = .51) nor for sad faces (*b* = −.01, *p* = .57) were significantly different from zero, according to simple slopes analyses.

Preliminary support for H5 applying to RT came, again, after splitting the data by country. The Emotion × Attitude interaction was significant in the Austrian, *b* = .01, *t*(65.16) = 2.36, *p* = .02, but not in the Turkish sample, *b* = .00, *t*(64.25) = 2.08, *p* = .86, also after excluding one third of trials from the Austrian sample.

### Faces with and without the hijab, Turkish sample

Next, we compared responses to faces with and without the hijab in the Turkish sample alone. The GLMM on categorization errors resulted in significant effects of Attitude (*b* = .26, *z* = 2.62, *p* = .009), and Emotion × Intensity × Cover (*b* = .12, *z* = 2.62, *p* = .009), and in a non-significant Intensity × Attitude interaction (*b* = .09, *z* = 1.82, *p* = .07). The three-way interaction reflected more categorisation errors for low-intensity happy faces with the hijab than the oval mask, and the reverse for low-intensity sad faces (Figure 6a-b).

The LMM on AUC of correct responses resulted in a significant Emotion × Intensity × Cover interaction, *b* = .02, *t*(19920) = 2.97, *p* = .003. The pattern of larger AUC to happy than sad low-intensity faces was more prevalent when categorising faces with the hijab than with an oval mask (Figure 6c–d).

The LMM on RT of correct responses resulted in significant effects of Emotion × Cover, *b* = −.01, *t*(61.94) = −3.60, *p* < .001, and Emotion × Intensity × Cover, *b*. 004, *t*(18930) = 2.66, *p* = .008. The three-way interaction reflects (Figure 6e-f) slower RTs to happy than sad low-intensity faces, the difference being larger for faces wearing the hijab, compared with faces with the oval mask.

## Discussion

To investigate how cultural and religious implicit biases influence emotion perception, Austrian and Turkish participants categorised, using the computer mouse in two separate experiments, happy and sad faces of women, shown with five levels of emotional intensity, and framed either by a head veil (hijab), or (in the Turkish sample) by an oval-shaped mask. Participants’ attitude towards the hijab were measured per questionnaire. In the following, we present and discuss first the results for trials in which faces with the hijab were shown to both samples, and then the outcome of comparing trials with and without the hijab in the Turkish sample only.

Cross-cultural comparison of responses to female faces with the hijab showed, across all three dependent variables (errors, AUC, RT), that participants with a more negative explicit attitude towards the hijab were biased in their implicit responses to faces with the hijab, especially for low-intensity happiness, and that this link was stronger in the Austrian sample.

**Figure 5.**
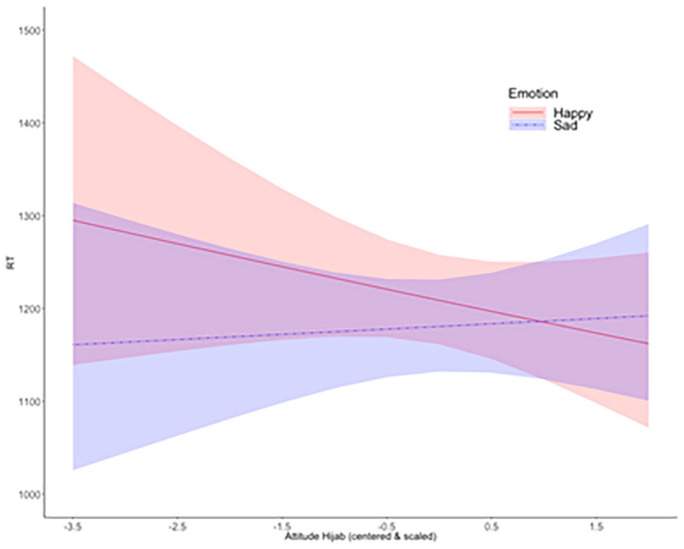
A more negative attitude towards the hijab resulted in slower RTs to happy but not sad faces, as indicated by a significant Emotion × Attitude interaction, which was also significant in the Austrian sample alone, but not in the Turkish sample alone. Lines indicate model fits, coloured areas the 95% confidence intervals.

Confirming H2, participants who explicitly reported a more negative attitude towards the hijab miscategorised more often happy female faces with the hijab, labelling them as sad (Figure 3). Confirming H3, the AUC in correct trials with happy faces was larger for participants with a more negative attitude towards the hijab, reflecting greater deflection of the mouse trajectory towards the “sad” label (Figure 4). This effect was somewhat larger for low-amplitude happiness (H6), although it should be noted that the Emotion × Intensity × Attitude interaction was not significant (*p* = .055). Confirming H4, RTs in correct trials with happy compared with sad faces were slower for participants with a more negative attitude towards the hijab (Figure 5). The described effects in all three dependent variables were significant in the Austrian, but absent in the Turkish sample, as indicated by analyses carried out after splitting the data by country. This suggests (H5) that the link between explicit attitudes towards the hijab and implicit emotion recognition bias, i.e., miscategorisations of happy faces, is stronger in the Austrian sample.

The finding that Austrians are biased to perceive happy faces with the hijab as sad is in line with previous research investigating Westerners’ emotion recognition biases in response to faces wearing Islamic head covers (Kret & de Gelder, 2012; Kret & Fischer, 2018), as well as to faces displaying other outgroup features, such as skin colour (Bijlstra et al., 2014). It is interesting to note that the here reported misattribution of sadness to faces with the hijab was observed despite the generally greater ease in recognising happy faces. Indeed, according to the “happy face advantage,” happy faces are typically recognised faster and more accurately, compared with other emotions (Calvo & Lundqvist, 2008; Palermo & Coltheart, 2004; Tottenham et al., 2009). Our results cannot be explained by eventual differences between emotions induced through the morphing, as all faces (neutral, happy, sad) featured a closed mouth, and morphing was thus of the same quality for happy and sad faces. Importantly, the present results also extend previous work by indicating that (1) *explicit* attitudes towards the hijab can predict *implicit* emotion recognition bias; (2) emotion recognition bias is not limited to faces wearing the niqab, but instead applies also to faces wearing the more revealing hijab; and (3) these biases emerge prevalently for weaker, ambiguous facial expressions. Possibly, these findings are caused by the activation of a perceptual set in our participants, i.e., stimulus-preceding mental associations between the hijab and negative stereotypes about Islam, and/or the concept that women wearing the hijab are oppressed and unfree (Allport, 1955; Dunning & Balcetis, 2013).

In contrast, no link between emotion recognition bias and the explicit attitude towards the hijab was found in the Turkish sample, as suggested by exploratory analyses after splitting the data by country. This may seem surprising, as Austrian and Turkish participants reported nearly identical explicit attitudes towards the hijab (Figure 2, H1 not confirmed), as well as similar behavioural responses across all three dependent variables (see Table S1 in the Supplementary Material).

Importantly, the lack of a significant Emotion × Attitude interaction in the Turkish sample is not due to a smaller number of trials showing faces with the hijab, as random exclusion of one third of trials did not change results in the Austrian sample. It is possible, however, that a significant Emotion × Attitude interaction would have emerged in a larger sample of Turkish participants. Indeed, our sensitivity power analysis (see Supplementary Material) suggests that statistical power was sufficient to detect the interaction in the Austrian and in the overall sample, but that this effect was underpowered in the Turkish sample, where a considerably larger number of participants (*N* > 800) would have been required for a significant Emotion × Attitude × Country interaction. Based on the results of our analyses by country, as well as the power analyses with data simulations, we conclude that the effects of explicit negative attitude towards the hijab onto emotion categorization biases in Turkish participants are weak at best (estimated effect size 0.23), while they are twice as large and thus clear in the Austrian sample (effect size 0.44).

A possible interpretation of these cross-cultural differences is that the Turkish participants tested in this study, who were young students mostly living in the capital, may not have been fully transparent in reporting their attitude towards the hijab. Indeed, compared with Austria, Turkey can be described as a “tight” culture, meaning that it is perceived as having more and stronger norms, from which deviance is less tolerated (Gelfand et al., 2011). Open declarations of non-religiosity are also becoming increasingly rare in Turkey (Demmrich & Blume, 2018; Sevinç et al., 2015). Thus, it is possible that at least a number of the Turkish participants may have felt critical about the hijab, but were reluctant to openly express this view. On the other hand, Austrian participants, while having a generally positive attitude towards the hijab, might have been less fearful to express their views to the matter.

**Figure 6.**
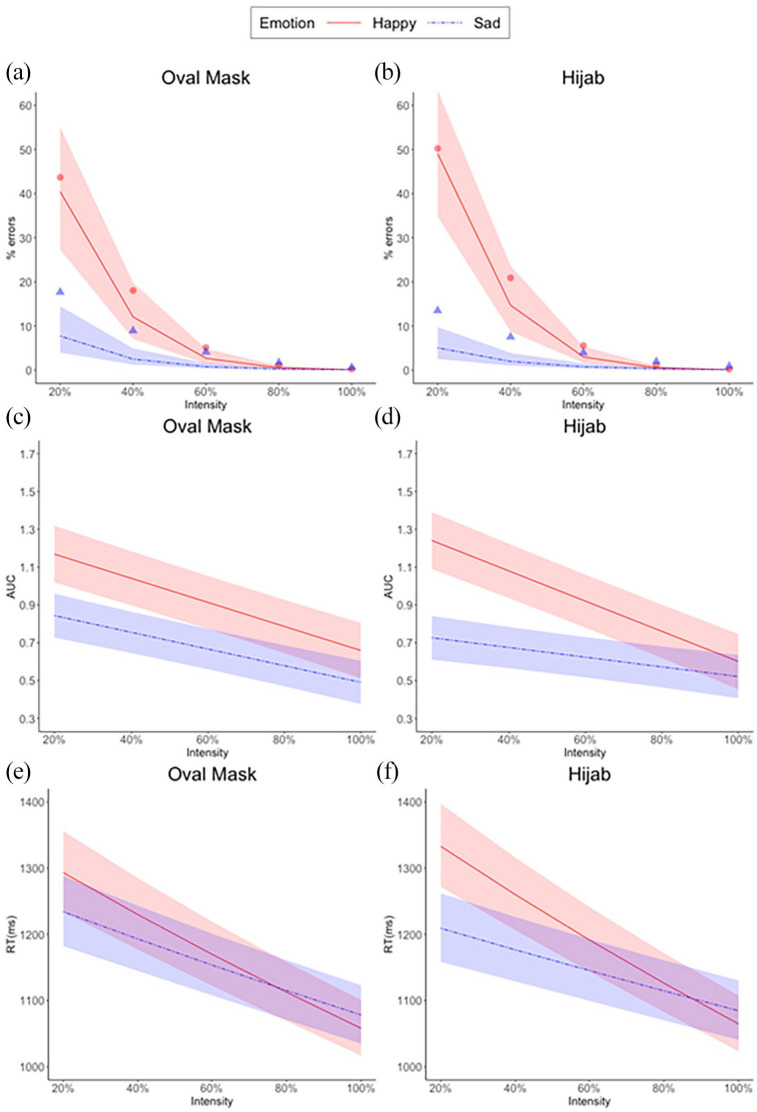
In Turkish participants, faces wearing a hijab resulted in more categorisation errors (b), greater AUC (d), and slower RT (f) at lower levels of emotional intensity and compared with faces with an oval mask (a, c, e). Lines indicate model fits, shaded areas the 95% CIs. Plots a and b also show marginal means, averaged at each level of intensity, for happy (red circles), and sad faces (blue triangles).

A negative implicit bias in response to faces with the hijab was nevertheless found in Turkish participants, even though it was unrelated to their explicit attitude towards the hijab. This was suggested from analyses comparing responses to female faces with and without the hijab. A significant Emotion × Intensity × Cover interaction was found for all three dependent variables (Figure 6), reflecting greater sadness attributed to mildly happy faces of women with the hijab, than without. Specifically, Turkish participants made more mistakes in categorising facial expressions of happiness in women with the hijab. Moreover, their mouse trajectory in correct trials showed greater curvature towards the sadness label, and their responses were slower, compared with faces in which the head contours were covered by an oval mask. In line with this, a significant two-way Emotion × Cover interaction was also found for RTs, reflecting slower correct responses to happy faces with the hijab than with the oval mask.

These behavioural results suggest that the hijab is associated—either directly, or indirectly through the association with negative stereotypes and prejudices—with sadness or other negative affective states in Turkish, as well as in Austrian participants. Moreover, the lack of a significant main effect of the factor Cover (hijab, oval mask) in the Turkish sample suggests that emotion recognition was not overall more difficult for faces with than without the hijab.

An alternative interpretation of the results exists, however. Female faces are often perceived as displaying more sadness (and fear and happiness) than male faces (Plant et al., 2004). Therefore, the hijab, which is exclusively worn by women, could have activated the concept of sadness by strengthening the concept of femaleness. This possibility could be tested in future research, by including as stimuli faces with the hijab and two types of negative facial expressions, e.g., sadness and anger.

This study tested participants from Austria and Turkey, to allow a direct comparison between countries that differ in their general prevalence and socio-cultural acceptance of the hijab. Indeed, Emotion × Attitude interactions were found in the Austrian, but not in the Turkish sample. However, the three-way Emotion × Attitude × Country interaction was not significant, due to insufficient sample size given the complexity of the model (see sensitivity power analyses in Supplementary Material). Alternatively, the interaction by country did not emerge when fitting the large model due to testing of university students from two large capital cities (Vienna and Ankara), which tend to be educated, industrialised, rich, and democratic (Henrich et al., 2010), and which might have had similar religious and political views in both countries (but see Table 1). The strong similarity of the Austrian and Turkish samples in these and other points allows to rule out the importance of their contribution to the results, and therefore constitutes a strength of the study. Indeed, in addition to the nearly identical distribution of explicit attitudes towards the hijab, the two groups were also similar in age, gender distribution, level of education, and type of studies. Importantly, despite their similarities on so many levels, the two groups of participants nevertheless differed in whether their explicit attitude towards the hijab predicted attribution of sadness to happy faces (Austria), or not (Turkey).

A limitation of the study is the lack of a proper control condition in the Austrian sample, who only categorised happy and sad faces with the hijab. It therefore cannot be excluded that Austrians with more negative attitudes towards the hijab also had the general tendency to perceive all faces as sadder. This seems unlikely, however, as the number of errors and the RT of correct categorisations (as well as the AUC after correction for multiple comparisons) in response to faces with the hijab did not differ significantly between Austrian and Turkish participants (see Table S1). This was neither the case for happy faces, nor for sad faces. Nevertheless, the findings should be interpreted with caution, and a replication of the study with a control condition in both participant groups is necessary, before firm conclusions can be made. Another limitation is that we only included the emotions happiness and sadness, while leaving out other relevant ones, such as fear, anger, and shame. This choice was taken in order not to make completion of the mousetracker task, which works best with two alternative choices, too long. Future studies should however investigate the link between explicit attitudes towards the hijab and implicit perception of other emotional facial expressions.

## Conclusion

A bias to perceive sadness in happy female faces with the hijab was found in both Austrian and Turkish participants. Explicit attitude towards the hijab predicted implicit emotion recognition bias in all participants, but in Austrians in particular. Attitude scores in Turkish participants had identical distribution but did not predict recognition bias. Nevertheless, Turkish participants attributed more sadness to happy faces with than without the hijab, suggesting that the hijab is associated with sadness or other negative emotions. These findings contribute to the understanding of how cultural and religious differences can complicate interpersonal communication.

## Supplemental Material

sj-docx-1-qjp-10.1177_17470218211048317 – Supplemental material for Emotion perception bias associated with the hijab in Austrian and Turkish participants Click here for additional data file. Supplemental material, sj-docx-1-qjp-10.1177_17470218211048317 for Emotion perception bias associated with the hijab in Austrian and Turkish participants by Sebastian Korb, Tugba Ceren Deniz, Bengi Ünal, Alasdair Clarke and Giorgia Silani in Quarterly Journal of Experimental Psychology

## Notes

1. Inclusion of these participants did not alter the pattern of results.

2. Faces 1, 3, 5, 6, 7, 8, 9, 18 were used for the task, face 13 for the practice trials.

3. Unless the model did not converge or resulted in singular fits, in which case the random effects structure was gradually simplified (e.g., removing the slope for the interaction). For model details, see tables in supplementary materials.

4. Full model tables in the Supplementary Material.
