# Supplementary material for: Emotion perception bias associated with the hijab in Austrian and Turkish participants
Source: Q J Exp Psychol (Hove). 2021 Sep 29;75(5):796–807. doi: 10.1177/17470218211048317 (PMC8958558; doi:10.1177/17470218211048317)
Supplement: sj-docx-1-qjp-10.1177_17470218211048317 – Supplemental material for Emotion perception bias associated with the hijab in Austrian and Turkish participants [file sj-docx-1-qjp-10.1177_17470218211048317.docx]

Supplementary material for

**Emotion perception bias associated with the hijab in Austrian and Turkish participants** Sebastian Korb, Tugba Ceren Deniz, Bengi ünal, Alasdair Clarke, Giorgia Silani

**Questionnaire**

To measure participants’ explicit attitude towards the hijab. Original in German/Turkish, responses on a 7-point Likert scale from 1 (Strongly disagree) to 7 (Strongly agree). Questions 4, 6, 7, and 8 were reverse coded. All questions were added to obtain participants’ attitude towards the hijab, with higher scores indicating a more positive attitude, and lower scores indicating a more negative attitude.

**Below are some statements that are used by people to describe themselves. Read through each statement and mark how much you agree with it selecting the point on the scale next to it. There are no right or wrong answers. Do not think too long, it is about your first impression. It is important to indicate how you generally feel.**

My attitude toward women who wear the Islamic headdress (hijab) is one of

1. Acceptance
2. Admiration
3. Affection
4. Antipathy
5. Approval
6. Contempt
7. Disapproval
8. Hostility
9. Sympathy

In my opinion, women wearing an Islamic headdress (hijab) are generally

1. Warm

**Table S1**: mean (SD) for Austrian and Turkish participants across the three dependent variables (DV). Values for *t* and *p*, resulting from linear regressions and uncorrected posthoc tests, are also provided.

| **DV** | **Emotion** | **Intensity** | **Austria** | **Turkey** | ***t (df)*** | ***p*** |
| --- | --- | --- | --- | --- | --- | --- |
| % errors | happy | 20 | 51.53 (25.54) | 52.95 (25.75) | -.65 (1390) | .52 |
|  | happy | 40 | 18.72 (20.06) | 17.23 (14.8) | .68 (1390) | .50 |
|  | happy | 60 | 3.35 (7.2) | 5.27 (7.5) | -.87 (1390) | .38 |
|  | happy | 80 | 1.88 (5.71) | 1.34 (3.17) | .25 (1390) | .81 |
|  | happy | 100 | 1.35 (4.09) | 1.25 (2.73) | .05 (1390) | .96 |
|  | sad | 20 | 15.61 (18.78) | 14.91 (21.29) | .32 (1390) | .75 |
|  | sad | 40 | 6.1 (9.96) | 6.25 (14.43) | -.06 (1390) | .95 |
|  | sad | 60 | 2.29 (4.92) | 3.3 (7.27) | -.46 (1390) | .64 |
|  | sad | 80 | 1.47 (4.98) | 1.61 (5.29) | -.06 (1390) | .94 |
|  | sad | 100 | 1.12 (4.4) | 1.88 (6.76) | -.35 (1390) | .73 |
| AUC | happy | 20 | 1.22 (1.02) | 1.46 (1.07) | -2.32 (1387) | .02 * |
|  | happy | 40 | 0.88 (0.56) | 1.14 (0.88) | -2.49 (1387) | .01 * |
|  | happy | 60 | 0.62 (0.42) | 0.73 (0.51) | -.96 (1387) | .33 |
|  | happy | 80 | 0.53 (0.41) | 0.74 (0.59) | -2.07 (1387) | .04 * |
|  | happy | 100 | 0.51 (0.36) | 0.70 (0.62) | -1.79 (1387) | .07 |
|  | sad | 20 | 0.8 (0.74) | 0.84 (0.84) | -.34 (1387) | .73 |
|  | sad | 40 | 0.57 (0.53) | 0.64 (0.45) | -.66 (1387) | .51 |
|  | sad | 60 | 0.52 (0.47) | 0.58 (0.48) | -.57 (1387) | .56 |
|  | sad | 80 | 0.48 (0.52) | 0.60 (0.49) | -1.10 (1387) | .27 |
|  | sad | 100 | 0.43 (0.44) | 0.54 (0.47) | -1.11 (1387) | .27 |
| RT | happy | 20 | 1445.21 (262.39) | 1493.58 (334.21) | -1.34 (1382) | .18 |
|  | happy | 40 | 1335.13 (214.84) | 1334.52 (275.54) | .02 (1382) | .99 |
|  | happy | 60 | 1224.56 (196.43) | 1183.44 (199.29) | 1.16 (1382) | .24 |
|  | happy | 80 | 1164.09 (175.75) | 1138.03 (189.92) | .73 (1382) | .46 |
|  | happy | 100 | 1152.11 (179.75) | 1140.32 (171.06) | .33 (1382) | .74 |
|  | sad | 20 | 1350.79 (208.02) | 1289.08 (229.19) | 1.74 (1382) | .08 |
|  | sad | 40 | 1251.79 (179.07) | 1222.21 (231.18) | .84 (1382) | .40 |
|  | sad | 60 | 1192.79 (159.1) | 1163.68 (197.98) | .82 (1382) | .41 |
|  | sad | 80 | 1174.22 (162.68) | 1153.75 (194.25) | .58 (1382) | .56 |
|  | sad | 100 | 1154.21 (165.57) | 1136.52 (195.4) | .50 (1382) | .62 |

**Sensitivity Power Analyses**

Three types of sensitivity power analyses were conducted on categorization errors^[[1]](#footnote-1)^. First, we simulated 100 samples of N = 141 and fitted to each the same LMM as described in the Analysis section (excluding the 3- and 4-way interactions)^[[2]](#footnote-2)^. The effect size of the Emotion X Attitude interaction was set to either 0, 0.2, or 0.25. The proportion of simulations that resulted in a significant (*p* < .05) Emotion X Attitude interaction (a measure of power) was, respectively, 10, 70, and 90%. Since the effect size estimated from the real data was 0.33 (see above and model table in Supplementary Material), our design appears sufficiently powered to detect this effect.

Second, we simulated 100 samples of N = 141 based on the full model (including 3- and 4-way interactions)^[[3]](#footnote-3)^, and fitted the LMM model (without Country) to Austrian and Turkish participants separately, after fixing the effect size of the Emotion X Attitude interaction to 0.2 or 0.4. This resulted, respectively, in 41 and 89% of significant interactions for the Austrian group, and in 30 and 77% for the Turkish group. Since the effect sizes estimated from the real data were 0.44 for the Austrian and 0.23 for the Turkish group (see above and model table in Supplementary Material), our study appears sufficiently powered to detect an Emotion X Attitude interaction in the Austrian sample (where power was > 0.89), but not in the Turkish sample (where power was ~ 0.3).

Third, we also ran simulations with increasing numbers of participants to find out at which N a sufficiently large power would be attained to adequately detect a hypothesized 3-way interaction between Emotion X Attitude X Country. To do so, we simulated 20 datasets for each of the following sample sizes: 142, 284, 426, 568, and 710. We fitted the full LMM (including all higher-order interactions) and calculated the proportion of times the 3-way interaction of interest was significant (*p* < .05). Even when five times more data were simulated (N = 710), a significant effect for the 3-way interaction was only found in 15 out of 20 times (corresponding to a power of 0.75).

It can be concluded, that statistical power was sufficient to detect an Emotion X Attitude interaction in the Austrian and the overall sample, but that this effect was underpowered in the Turkish sample, and that a much larger sample size (N = 800 or more) would have be required for a significant Emotion X Attitude X Country interaction.

| **Emotion Categorisation Errors for faces with Hijab** | | | | | | | | | | | | |
| --- | --- | --- | --- | --- | --- | --- | --- | --- | --- | --- | --- | --- |
|  | **All** | | | | **Austria** | | | | **Turkey** | | | |
| *Predictors* | *Log-Odds* | *CI* | *Statistic* | *p* | *Log-Odds* | *CI* | *Statistic* | *p* | *Log-Odds* | *CI* | *Statistic* | *p* |
| Intercept | -5.35 | -6.03 – -4.67 | -15.34 | **<0.001** | -5.36 | -6.03 – -4.69 | -15.64 | **<0.001** | -5.45 | -6.34 – -4.56 | -12.01 | **<0.001** |
| Emotion | -0.53 | -1.31 – 0.24 | -1.34 | 0.179 | -0.34 | -1.19 – 0.50 | -0.80 | 0.426 | -0.78 | -1.62 – 0.07 | -1.80 | 0.071 |
| Intensity | -2.76 | -3.18 – -2.34 | -12.86 | **<0.001** | -2.84 | -3.26 – -2.43 | -13.44 | **<0.001** | -2.74 | -3.28 – -2.20 | -10.00 | **<0.001** |
| Attitude | 0.06 | -0.21 – 0.34 | 0.44 | 0.661 | -0.16 | -0.53 – 0.22 | -0.83 | 0.408 | 0.28 | -0.14 – 0.71 | 1.32 | 0.186 |
| Country | 0.08 | -0.19 – 0.35 | 0.57 | 0.569 |  |  |  |  |  |  |  |  |
| Emotion X Intensity | 0.68 | 0.56 – 0.80 | 10.85 | **<0.001** | 0.75 | 0.58 – 0.91 | 8.81 | **<0.001** | 0.61 | 0.42 – 0.79 | 6.49 | **<0.001** |
| Emotion X Attitude | 0.33 | 0.03 – 0.63 | 2.12 | **0.034** | 0.44 | 0.05 – 0.84 | 2.18 | **0.029** | 0.23 | -0.23 – 0.69 | 0.98 | 0.326 |
| Intensity X Attitude | 0.06 | -0.11 – 0.23 | 0.68 | 0.499 | -0.02 | -0.26 – 0.21 | -0.20 | 0.841 | 0.14 | -0.11 – 0.38 | 1.11 | 0.269 |
| Emotion X Country | -0.13 | -0.42 – 0.17 | -0.83 | 0.404 |  |  |  |  |  |  |  |  |
| Intensity X Country | 0.11 | -0.05 – 0.28 | 1.32 | 0.185 |  |  |  |  |  |  |  |  |
| Attitude X Country | 0.21 | -0.06 – 0.49 | 1.51 | 0.132 |  |  |  |  |  |  |  |  |
| Emotion X Intensity X Attitude | 0.01 | -0.09 – 0.12 | 0.24 | 0.809 | 0.06 | -0.08 – 0.20 | 0.89 | 0.376 | -0.03 | -0.19 – 0.13 | -0.36 | 0.718 |
| Emotion X Intensity X Country | -0.05 | -0.14 – 0.05 | -0.89 | 0.372 |  |  |  |  |  |  |  |  |
| Emotion X Attitude X Country | -0.12 | -0.43 – 0.18 | -0.79 | 0.431 |  |  |  |  |  |  |  |  |
| Intensity X Attitude X Country | 0.08 | -0.09 – 0.25 | 0.92 | 0.355 |  |  |  |  |  |  |  |  |
| Emotion X Intensity X Attitude X Country | -0.06 | -0.16 – 0.05 | -1.06 | 0.291 |  |  |  |  |  |  |  |  |
| **Random Effects** | | | | | | | | | | | | |
| σ^2^ | 3.29 | | | | 3.29 | | | | 3.29 | | | |
| τ_00_ | 1.98 _subject_ | | | | 1.94 _subject_ | | | | 2.21 _subject_ | | | |
|  | 0.79 _face_id_ | | | | 0.59 _face_id_ | | | | 1.20 _face_id_ | | | |
| τ_11_ | 2.57 _subject.emotion_C1_ | | | | 2.30 _subject.emotion_C1_ | | | | 2.95 _subject.emotion_C1_ | | | |
|  | 0.64 _subject.intensity_C_ | | | | 0.68 _subject.intensity_C_ | | | | 0.60 _subject.intensity_C_ | | | |
|  | 1.05 _face_id.emotion_C1_ | | | | 1.14 _face_id.emotion_C1_ | | | | 0.99 _face_id.emotion_C1_ | | | |
|  | 0.29 _face_id.intensity_C_ | | | | 0.21 _face_id.intensity_C_ | | | | 0.44 _face_id.intensity_C_ | | | |
| ρ_01_ | 0.36 | | | | 0.16 | | | | 0.57 | | | |
|  | 0.97 | | | | 0.97 | | | | 0.97 | | | |
|  | -0.58 | | | | -0.55 | | | | -0.55 | | | |
|  | 0.76 | | | | 0.67 | | | | 0.84 | | | |
| ICC | 0.69 | | | | 0.68 | | | | 0.72 | | | |
| N | 141 _subject_ | | | | 71 _subject_ | | | | 70 _subject_ | | | |
|  | 8 _face_id_ | | | | 8 _face_id_ | | | | 8 _face_id_ | | | |
| Observations | 28240 | | | | 17040 | | | | 11200 | | | |
| Marginal R^2^/ Conditional R^2^ | 0.451 / 0.830 | | | | 0.469 / 0.828 | | | | 0.425 / 0.838 | | | |

| **Emotion Categorisation Errors for faces with Hijab (similar number of trials in both countries)** | | | | | | | | |
| --- | --- | --- | --- | --- | --- | --- | --- | --- |
|  | **Austria (2/3 of trials)** | | | | **Turkey** | | | |
| *Predictors* | *Log-Odds* | *CI* | *Statistic* | *p* | *Log-Odds* | *CI* | *Statistic* | *p* |
| Intercept | -5.95 | -6.79 – -5.11 | -13.92 | **<0.001** | -5.45 | -6.34 – -4.56 | -12.01 | **<0.001** |
| Emotion | -0.40 | -1.29 – 0.50 | -0.87 | 0.383 | -0.78 | -1.62 – 0.07 | -1.80 | 0.071 |
| Intensity | -3.34 | -3.95 – -2.73 | -10.67 | **<0.001** | -2.74 | -3.28 – -2.20 | -10.00 | **<0.001** |
| Attitude | -0.03 | -0.49 – 0.43 | -0.14 | 0.885 | 0.28 | -0.14 – 0.71 | 1.32 | 0.186 |
| Emotion X Intensity | 0.79 | 0.57 – 1.02 | 6.86 | **<0.001** | 0.61 | 0.42 – 0.79 | 6.49 | **<0.001** |
| Emotion X Attitude | 0.66 | 0.21 – 1.10 | 2.86 | **0.004** | 0.23 | -0.23 – 0.69 | 0.98 | 0.326 |
| Intensity X Attitude | 0.08 | -0.23 – 0.39 | 0.51 | 0.607 | 0.14 | -0.11 – 0.38 | 1.11 | 0.269 |
| Emotion X Intensity X Attitude | 0.15 | -0.05 – 0.35 | 1.49 | 0.136 | -0.03 | -0.19 – 0.13 | -0.36 | 0.718 |
| **Random Effects** | | | | | | | | |
| σ^2^ | 3.29 | | | | 3.29 | | | |
| τ_00_ | 2.65 _subject_ | | | | 2.21 _subject_ | | | |
|  | 0.88 _face_id_ | | | | 1.20 _face_id_ | | | |
| τ_11_ | 2.46 _subject.emotion_C1_ | | | | 2.95 _subject.emotion_C1_ | | | |
|  | 1.04 _subject.intensity_C_ | | | | 0.60 _subject.intensity_C_ | | | |
|  | 1.19 _face_id.emotion_C1_ | | | | 0.99 _face_id.emotion_C1_ | | | |
|  | 0.52 _face_id.intensity_C_ | | | | 0.44 _face_id.intensity_C_ | | | |
| ρ_01_ | 0.13 | | | | 0.57 | | | |
|  | 0.94 | | | | 0.97 | | | |
|  | -0.47 | | | | -0.55 | | | |
|  | 0.90 | | | | 0.84 | | | |
| ICC | 0.73 | | | | 0.72 | | | |
| N | 71 _subject_ | | | | 70 _subject_ | | | |
|  | 8 _face_id_ | | | | 8 _face_id_ | | | |
| Observations | 10650 | | | | 11200 | | | |
| Marginal R^2^/ Conditional R^2^ | 0.508 / 0.865 | | | | 0.425 / 0.838 | | | |

| **AUC for faces with Hijab (correct trials only)** | | | | | | | | | | | | |
| --- | --- | --- | --- | --- | --- | --- | --- | --- | --- | --- | --- | --- |
|  | **All** | | | | **Austria** | | | | **Turkey** | | | |
| *Predictors* | *Estimates* | *CI* | *Statistic* | *p* | *Estimates* | *CI* | *Statistic* | *p* | *Estimates* | *CI* | *Statistic* | *p* |
| Intercept | 0.72 | 0.57 – 0.87 | 9.47 | **<0.001** | 0.62 | 0.54 – 0.70 | 15.16 | **<0.001** | 0.76 | 0.66 – 0.86 | 14.60 | **<0.001** |
| Emotion | -0.17 | -0.37 – 0.03 | -1.65 | 0.099 | -0.08 | -0.16 – 0.01 | -1.81 | 0.071 | -0.14 | -0.26 – -0.03 | -2.55 | **0.011** |
| Intensity | -0.23 | -0.28 – -0.18 | -8.52 | **<0.001** | -0.16 | -0.19 – -0.13 | -9.96 | **<0.001** | -0.15 | -0.18 – -0.12 | -8.82 | **<0.001** |
| Attitude | -0.08 | -0.19 – 0.04 | -1.35 | 0.177 | 0.04 | -0.03 – 0.12 | 1.08 | 0.282 | -0.05 | -0.14 – 0.04 | -1.12 | 0.261 |
| Country | 0.20 | 0.04 – 0.37 | 2.40 | **0.017** |  |  |  |  |  |  |  |  |
| Emotion X Intensity | 0.12 | 0.03 – 0.20 | 2.69 | **0.007** | 0.05 | 0.03 – 0.07 | 5.91 | **<0.001** | 0.08 | 0.06 – 0.10 | 6.98 | **<0.001** |
| Emotion X Attitude | 0.24 | 0.09 – 0.38 | 3.23 | **0.001** | 0.12 | 0.05 – 0.18 | 3.53 | **<0.001** | 0.06 | -0.02 – 0.14 | 1.55 | 0.121 |
| Intensity X Attitude | 0.03 | -0.01 – 0.08 | 1.39 | 0.164 | 0.01 | -0.02 – 0.03 | 0.42 | 0.677 | -0.00 | -0.03 – 0.03 | -0.23 | 0.820 |
| Emotion X Country | -0.13 | -0.34 – 0.08 | -1.21 | 0.228 |  |  |  |  |  |  |  |  |
| Intensity X Country | -0.02 | -0.08 – 0.05 | -0.55 | 0.582 |  |  |  |  |  |  |  |  |
| Attitude X Country | -0.03 | -0.20 – 0.13 | -0.41 | 0.681 |  |  |  |  |  |  |  |  |
| Emotion X Intensity X Attitude | -0.05 | -0.11 – 0.00 | -1.94 | 0.053 | -0.03 | -0.04 – -0.01 | -3.07 | **0.002** | -0.01 | -0.03 – 0.02 | -0.62 | 0.538 |
| Emotion X Intensity X Country | 0.05 | -0.03 – 0.13 | 1.28 | 0.200 |  |  |  |  |  |  |  |  |
| Emotion X Attitude X Country | -0.11 | -0.32 – 0.10 | -1.06 | 0.290 |  |  |  |  |  |  |  |  |
| Intensity X Attitude X Country | -0.03 | -0.10 – 0.04 | -0.90 | 0.369 |  |  |  |  |  |  |  |  |
| Emotion X Intensity X Attitude X Country | 0.04 | -0.04 – 0.12 | 1.02 | 0.308 |  |  |  |  |  |  |  |  |
| **Random Effects** | | | | | | | | | | | | |
| σ^2^ | 1.09 | | | | 1.00 | | | | 1.25 | | | |
| τ_00_ | 0.24 _subject_ | | | | 0.10 _subject_ | | | | 0.13 _subject_ | | | |
|  | 0.02 _face_id_ | | | | 0.00 _face_id_ | | | | 0.01 _face_id_ | | | |
| τ_11_ | 0.37 _subject.emotion_C1_ | | | | 0.08 _subject.emotion_C1_ | | | | 0.10 _subject.emotion_C1_ | | | |
|  | 0.02 _subject.intensity_C_ | | | | 0.01 _subject.intensity_C_ | | | | 0.01 _subject.intensity_C_ | | | |
|  | 0.03 _subject.emotion_C1:intensity_C_ | | | | 0.01 _face_id.emotion_C1_ | | | | 0.01 _face_id.emotion_C1_ | | | |
|  | 0.04 _face_id.emotion_C1_ | | | | 0.00 _face_id.intensity_C_ | | | | 0.00 _face_id.intensity_C_ | | | |
|  | 0.00 _face_id.intensity_C_ | | | |  | | | |  | | | |
|  | 0.01 _face_id.emotion_C1:intensity_C_ | | | |  | | | |  | | | |
| ρ_01_ | -0.70 | | | | 0.14 | | | | -0.29 | | | |
|  | -0.55 | | | | -0.56 | | | | -0.35 | | | |
|  | 0.51 | | | | -0.01 | | | | -0.70 | | | |
|  | -0.93 | | | | 0.31 | | | | 0.61 | | | |
|  | -0.95 | | | |  | | | |  | | | |
|  | 0.96 | | | |  | | | |  | | | |
| ICC | 0.18 | | | | 0.16 | | | | 0.17 | | | |
| N | 141 _subject_ | | | | 71 _subject_ | | | | 70 _subject_ | | | |
|  | 8 _face_id_ | | | | 8 _face_id_ | | | | 8 _face_id_ | | | |
| Observations | 25291 | | | | 15278 | | | | 10013 | | | |
| Marginal R^2^/ Conditional R^2^ | 0.041 / 0.214 | | | | 0.037 / 0.194 | | | | 0.031 / 0.196 | | | |

| **AUC for faces with Hijab (correct trials only, similar number of trials in both countries)** | | | | | | | | |
| --- | --- | --- | --- | --- | --- | --- | --- | --- |
|  | **Austria (2/3 of trials)** | | | | **Turkey** | | | |
| *Predictors* | *Estimates* | *CI* | *Statistic* | *p* | *Estimates* | *CI* | *Statistic* | *p* |
| Intercept | 0.63 | 0.55 – 0.71 | 15.12 | **<0.001** | 0.76 | 0.66 – 0.86 | 14.60 | **<0.001** |
| Emotion | -0.08 | -0.17 – 0.02 | -1.56 | 0.119 | -0.14 | -0.26 – -0.03 | -2.55 | **0.011** |
| Intensity | -0.15 | -0.19 – -0.12 | -8.52 | **<0.001** | -0.15 | -0.18 – -0.12 | -8.82 | **<0.001** |
| Attitude | 0.06 | -0.01 – 0.14 | 1.61 | 0.107 | -0.05 | -0.14 – 0.04 | -1.12 | 0.261 |
| Emotion X Intensity | 0.05 | 0.03 – 0.07 | 5.16 | **<0.001** | 0.08 | 0.06 – 0.10 | 6.98 | **<0.001** |
| Emotion X Attitude | 0.10 | 0.03 – 0.18 | 2.73 | **0.006** | 0.06 | -0.02 – 0.14 | 1.55 | 0.121 |
| Intensity X Attitude | 0.01 | -0.03 – 0.04 | 0.43 | 0.668 | -0.00 | -0.03 – 0.03 | -0.23 | 0.820 |
| Emotion X Intensity X Attitude | -0.02 | -0.04 – -0.00 | -2.11 | **0.035** | -0.01 | -0.03 – 0.02 | -0.62 | 0.538 |
| **Random Effects** | | | | | | | | |
| σ^2^ | 0.97 | | | | 1.25 | | | |
| τ_00_ | 0.10 _subject_ | | | | 0.13 _subject_ | | | |
|  | 0.00 _face_id_ | | | | 0.01 _face_id_ | | | |
| τ_11_ | 0.10 _subject.emotion_C1_ | | | | 0.10 _subject.emotion_C1_ | | | |
|  | 0.01 _subject.intensity_C_ | | | | 0.01 _subject.intensity_C_ | | | |
|  | 0.01 _face_id.emotion_C1_ | | | | 0.01 _face_id.emotion_C1_ | | | |
|  | 0.00 _face_id.intensity_C_ | | | | 0.00 _face_id.intensity_C_ | | | |
| ρ_01_ | 0.10 | | | | -0.29 | | | |
|  | -0.49 | | | | -0.35 | | | |
|  | -0.16 | | | | -0.70 | | | |
|  | 0.57 | | | | 0.61 | | | |
| ICC | 0.19 | | | | 0.17 | | | |
| N | 71 _subject_ | | | | 70 _subject_ | | | |
|  | 8 _face_id_ | | | | 8 _face_id_ | | | |
| Observations | 9605 | | | | 10013 | | | |
| Marginal R^2^/ Conditional R^2^ | 0.036 / 0.218 | | | | 0.031 / 0.196 | | | |

| **RT for faces with Hijab (correct trials only)** | | | | | | | | | | | | |
| --- | --- | --- | --- | --- | --- | --- | --- | --- | --- | --- | --- | --- |
|  | **All** | | | | **Austria** | | | | **Turkey** | | | |
| *Predictors* | *Estimates* | *CI* | *Statistic* | *p* | *Estimates* | *CI* | *Statistic* | *p* | *Estimates* | *CI* | *Statistic* | *p* |
| Intercept | 7.10 | 7.06 – 7.14 | 349.18 | **<0.001** | 7.09 | 7.05 – 7.12 | 412.96 | **<0.001** | 7.05 | 7.02 – 7.09 | 360.02 | **<0.001** |
| Emotion | -0.02 | -0.06 – 0.02 | -1.18 | 0.239 | -0.01 | -0.03 – 0.01 | -1.23 | 0.219 | -0.02 | -0.04 – 0.00 | -1.56 | 0.119 |
| Intensity | -0.08 | -0.09 – -0.07 | -13.94 | **<0.001** | -0.06 | -0.07 – -0.06 | -18.94 | **<0.001** | -0.06 | -0.06 – -0.05 | -11.31 | **<0.001** |
| Attitude | -0.02 | -0.05 – 0.02 | -1.10 | 0.271 | -0.01 | -0.04 – 0.02 | -0.47 | 0.638 | 0.01 | -0.02 – 0.05 | 0.70 | 0.484 |
| Country | -0.02 | -0.07 – 0.03 | -0.90 | 0.367 |  |  |  |  |  |  |  |  |
| Emotion X Intensity | 0.03 | 0.01 – 0.05 | 3.06 | **0.002** | 0.02 | 0.01 – 0.02 | 3.18 | **0.001** | 0.02 | 0.01 – 0.02 | 8.24 | **<0.001** |
| Emotion X Attitude | 0.02 | 0.00 – 0.05 | 2.08 | **0.037** | 0.01 | 0.00 – 0.02 | 2.36 | **0.018** | 0.00 | -0.01 – 0.01 | 0.18 | 0.860 |
| Intensity X Attitude | -0.00 | -0.01 – 0.01 | -0.16 | 0.873 | 0.00 | -0.01 – 0.01 | 0.10 | 0.917 | 0.00 | -0.01 – 0.01 | 0.21 | 0.833 |
| Emotion X Country | -0.01 | -0.04 – 0.02 | -0.64 | 0.520 |  |  |  |  |  |  |  |  |
| Intensity X Country | -0.00 | -0.01 – 0.01 | -0.23 | 0.820 |  |  |  |  |  |  |  |  |
| Attitude X Country | 0.03 | -0.02 – 0.08 | 1.21 | 0.226 |  |  |  |  |  |  |  |  |
| Emotion X Intensity X Attitude | 0.00 | -0.01 – 0.01 | 0.52 | 0.600 | 0.00 | -0.00 – 0.01 | 0.66 | 0.508 | 0.00 | -0.00 – 0.00 | 0.07 | 0.947 |
| Emotion X Intensity X Country | 0.01 | -0.00 – 0.02 | 1.37 | 0.171 |  |  |  |  |  |  |  |  |
| Emotion X Attitude X Country | -0.02 | -0.06 – 0.01 | -1.30 | 0.192 |  |  |  |  |  |  |  |  |
| Intensity X Attitude X Country | 0.00 | -0.01 – 0.01 | 0.19 | 0.849 |  |  |  |  |  |  |  |  |
| Emotion X Intensity X Attitude X Country | -0.00 | -0.02 – 0.01 | -0.26 | 0.799 |  |  |  |  |  |  |  |  |
| **Random Effects** | | | | | | | | | | | | |
| σ^2^ | 0.04 | | | | 0.04 | | | | 0.05 | | | |
| τ_00_ | 0.02 _subject_ | | | | 0.02 _subject_ | | | | 0.02 _subject_ | | | |
|  | 0.00 _face_id_ | | | | 0.00 _face_id_ | | | | 0.00 _face_id_ | | | |
| τ_11_ | 0.01 _subject.emotion_C1_ | | | | 0.00 _subject.emotion_C1_ | | | | 0.00 _subject.emotion_C1_ | | | |
|  | 0.00 _subject.intensity_C_ | | | | 0.00 _subject.intensity_C_ | | | | 0.00 _subject.intensity_C_ | | | |
|  | 0.00 _subject.emotion_C1:intensity_C_ | | | | 0.00 _subject.emotion_C1:intensity_C_ | | | | 0.00 _face_id.emotion_C1_ | | | |
|  | 0.00 _face_id.emotion_C1_ | | | | 0.00 _face_id.emotion_C1_ | | | | 0.00 _face_id.intensity_C_ | | | |
|  | 0.00 _face_id.intensity_C_ | | | | 0.00 _face_id.intensity_C_ | | | |  | | | |
|  | 0.00 _face_id.emotion_C1:intensity_C_ | | | | 0.00 _face_id.emotion_C1:intensity_C_ | | | |  | | | |
| ρ_01_ | -0.38 | | | | -0.11 | | | | -0.02 | | | |
|  | -0.26 | | | | 0.03 | | | | -0.24 | | | |
|  | 0.32 | | | | -0.01 | | | | 0.42 | | | |
|  | -0.64 | | | | 0.28 | | | | 0.50 | | | |
|  | -0.77 | | | | -0.29 | | | |  | | | |
|  | 0.84 | | | | 0.48 | | | |  | | | |
| ICC | 0.37 | | | | 0.36 | | | | 0.36 | | | |
| N | 141 _subject_ | | | | 71 _subject_ | | | | 70 _subject_ | | | |
|  | 8 _face_id_ | | | | 8 _face_id_ | | | | 8 _face_id_ | | | |
| Observations | 24455 | | | | 14835 | | | | 9620 | | | |
| Marginal R^2^/ Conditional R^2^ | 0.061 / 0.409 | | | | 0.067 / 0.406 | | | | 0.044 / 0.385 | | | |

| **RT for faces with Hijab (correct trials only, similar number of trials in both countries)** | | | | | | | | |
| --- | --- | --- | --- | --- | --- | --- | --- | --- |
|  | **Austria (2/3 of trials)** | | | | **Turkey** | | | |
| *Predictors* | *Estimates* | *CI* | *Statistic* | *p* | *Estimates* | *CI* | *Statistic* | *p* |
| Intercept | 7.08 | 7.05 – 7.12 | 413.58 | **<0.001** | 7.05 | 7.02 – 7.09 | 360.02 | **<0.001** |
| Emotion | -0.01 | -0.03 – 0.01 | -1.00 | 0.318 | -0.02 | -0.04 – 0.00 | -1.56 | 0.119 |
| Intensity | -0.06 | -0.07 – -0.06 | -30.04 | **<0.001** | -0.06 | -0.06 – -0.05 | -11.31 | **<0.001** |
| Attitude | -0.01 | -0.04 – 0.02 | -0.48 | 0.631 | 0.01 | -0.02 – 0.05 | 0.70 | 0.484 |
| Emotion X Intensity | 0.01 | 0.01 – 0.01 | 4.98 | **<0.001** | 0.02 | 0.01 – 0.02 | 8.24 | **<0.001** |
| Emotion X Attitude | 0.01 | 0.00 – 0.02 | 2.11 | **0.035** | 0.00 | -0.01 – 0.01 | 0.18 | 0.860 |
| Intensity X Attitude | 0.00 | -0.00 – 0.00 | 0.11 | 0.909 | 0.00 | -0.01 – 0.01 | 0.21 | 0.833 |
| Emotion X Intensity X Attitude | 0.00 | 0.00 – 0.01 | 2.34 | **0.020** | 0.00 | -0.00 – 0.00 | 0.07 | 0.947 |
| **Random Effects** | | | | | | | | |
| σ^2^ | 0.04 | | | | 0.05 | | | |
| τ_00_ | 0.02 _subject_ | | | | 0.02 _subject_ | | | |
|  | 0.00 _face_id_ | | | | 0.00 _face_id_ | | | |
| τ_11_ | 0.00 _subject.emotion_C1_ | | | | 0.00 _subject.emotion_C1_ | | | |
|  | 0.00 _face_id.emotion_C1_ | | | | 0.00 _subject.intensity_C_ | | | |
|  |  | | | | 0.00 _face_id.emotion_C1_ | | | |
|  |  | | | | 0.00 _face_id.intensity_C_ | | | |
| ρ_01_ | -0.11 _subject_ | | | | -0.02 | | | |
|  | 0.40 _face_id_ | | | | -0.24 | | | |
|  |  | | | | 0.42 | | | |
|  |  | | | | 0.50 | | | |
| ICC | 0.36 | | | | 0.36 | | | |
| N | 71 _subject_ | | | | 70 _subject_ | | | |
|  | 8 _face_id_ | | | | 8 _face_id_ | | | |
| Observations | 9333 | | | | 9620 | | | |
| Marginal R^2^/ Conditional R^2^ | 0.064 / 0.400 | | | | 0.044 / 0.385 | | | |

**Categorisation errors of faces with 2 cover types (hijab, oval mask) in Turkish sample**

|  | **error** | | | |
| --- | --- | --- | --- | --- |
| *Predictors* | *Log-Odds* | *CI* | *Statistic* | *p* |
| (Intercept) | -4.19 | -4.74 – -3.64 | -14.97 | **<0.001** |
| emotion_C [1] | -0.66 | -1.02 – -0.31 | -3.66 | **<0.001** |
| intensity_C | -1.93 | -2.02 – -1.84 | -40.87 | **<0.001** |
| att_hijab_C | 0.26 | 0.07 – 0.46 | 2.62 | **0.009** |
| cover_C [1] | 0.02 | -0.08 – 0.11 | 0.40 | 0.688 |
| emotion_C [1] * intensity_C | 0.41 | 0.32 – 0.50 | 8.98 | **<0.001** |
| emotion_C [1] * att_hijab_C | 0.21 | -0.15 – 0.56 | 1.15 | 0.249 |
| intensity_C * att_hijab_C | 0.09 | -0.01 – 0.18 | 1.82 | 0.069 |
| emotion_C [1] * cover_C [1] | -0.03 | -0.15 – 0.08 | -0.61 | 0.544 |
| intensity_C * cover_C [1] | 0.03 | -0.05 – 0.12 | 0.75 | 0.453 |
| att_hijab_C * cover_C [1] | -0.04 | -0.13 – 0.06 | -0.76 | 0.450 |
| (emotion_C [1] * intensity_C) * att_hijab_C | -0.05 | -0.15 – 0.04 | -1.11 | 0.268 |
| (emotion_C [1] * intensity_C) * cover_C [1] | 0.12 | 0.03 – 0.21 | 2.62 | **0.009** |
| (emotion_C [1] * att_hijab_C) * cover_C [1] | -0.02 | -0.11 – 0.08 | -0.39 | 0.696 |
| (intensity_C * att_hijab_C) * cover_C [1] | 0.01 | -0.08 – 0.10 | 0.18 | 0.856 |
| (emotion_C [1] * intensity_C * att_hijab_C) * cover_C [1] | 0.01 | -0.08 – 0.10 | 0.27 | 0.790 |
| **Random Effects** | | | | |
| σ^2^ | 3.29 | | | |
| τ_00_ _subject_ | 0.37 | | | |
| τ_00_ _face_id_ | 0.54 | | | |
| τ_11_ _subject.emotion_C1_ | 1.96 | | | |
| τ_11_ _subject.cover_C1_ | 0.00 | | | |
| ρ_01_ _subject.emotion_C1_ | 0.60 | | | |
| ρ_01_ _subject.cover_C1_ | -0.34 | | | |
| ICC | 0.47 | | | |
| N _subject_ | 70 | | | |
| N _face_id_ | 8 | | | |
| Observations | 22400 | | | |
| Marginal R^2^ / Conditional R^2^ | 0.421 / 0.691 | | | |

**Area under the curve for faces with 2 cover types (hijab, oval mask) in Turkish sample**

|  | **AUC_new** | | | |
| --- | --- | --- | --- | --- |
| *Predictors* | *Estimates* | *CI* | *Statistic* | *p* |
| (Intercept) | 0.76 | 0.67 – 0.86 | 15.78 | **<0.001** |
| emotion_C [1] | -0.13 | -0.21 – -0.05 | -3.28 | **0.001** |
| intensity_C | -0.15 | -0.16 – -0.13 | -18.12 | **<0.001** |
| att_hijab_C | -0.06 | -0.15 – 0.03 | -1.36 | 0.175 |
| cover_C [1] | -0.01 | -0.02 – 0.01 | -1.12 | 0.264 |
| emotion_C [1] * intensity_C | 0.05 | 0.03 – 0.07 | 6.26 | **<0.001** |
| emotion_C [1] * att_hijab_C | 0.06 | -0.02 – 0.13 | 1.44 | 0.149 |
| intensity_C * att_hijab_C | 0.00 | -0.01 – 0.02 | 0.57 | 0.568 |
| emotion_C [1] * cover_C [1] | -0.01 | -0.03 – 0.01 | -1.20 | 0.229 |
| intensity_C * cover_C [1] | 0.00 | -0.01 – 0.02 | 0.19 | 0.849 |
| att_hijab_C * cover_C [1] | 0.01 | -0.01 – 0.02 | 1.15 | 0.248 |
| (emotion_C [1] * intensity_C) * att_hijab_C | -0.01 | -0.02 – 0.01 | -0.98 | 0.325 |
| (emotion_C [1] * intensity_C) * cover_C [1] | 0.02 | 0.01 – 0.04 | 2.97 | **0.003** |
| (emotion_C [1] * att_hijab_C) * cover_C [1] | 0.01 | -0.01 – 0.02 | 0.79 | 0.428 |
| (intensity_C * att_hijab_C) * cover_C [1] | -0.01 | -0.02 – 0.01 | -0.91 | 0.361 |
| (emotion_C [1] * intensity_C * att_hijab_C) * cover_C [1] | 0.00 | -0.02 – 0.02 | 0.07 | 0.943 |
| **Random Effects** | | | | |
| σ^2^ | 1.26 | | | |
| τ_00_ _subject_ | 0.14 | | | |
| τ_00_ _face_id_ | 0.00 | | | |
| τ_11_ _subject.emotion_C1_ | 0.11 | | | |
| ρ_01_ _subject_ | -0.31 | | | |
| ICC | 0.16 | | | |
| N _subject_ | 70 | | | |
| N _face_id_ | 8 | | | |
| Observations | 20076 | | | |
| Marginal R^2^ / Conditional R^2^ | 0.028 / 0.183 | | | |

**RT for faces with 2 cover types (hijab, oval mask) in Turkish sample**

|  | **log(RT)** | | | |
| --- | --- | --- | --- | --- |
| *Predictors* | *Estimates* | *CI* | *Statistic* | *p* |
| (Intercept) | 7.05 | 7.01 – 7.09 | 360.78 | **<0.001** |
| emotion_C [1] | -0.01 | -0.02 – 0.00 | -1.72 | 0.086 |
| intensity_C | -0.06 | -0.06 – -0.05 | -19.43 | **<0.001** |
| att_hijab_C | 0.01 | -0.02 – 0.05 | 0.69 | 0.488 |
| cover_C [1] | 0.00 | -0.00 – 0.01 | 1.54 | 0.123 |
| emotion_C [1] * intensity_C | 0.02 | 0.01 – 0.02 | 6.36 | **<0.001** |
| emotion_C [1] * att_hijab_C | 0.00 | -0.01 – 0.02 | 0.47 | 0.640 |
| intensity_C * att_hijab_C | 0.00 | -0.00 – 0.01 | 0.40 | 0.688 |
| emotion_C [1] * cover_C [1] | -0.01 | -0.01 – -0.00 | -3.60 | **<0.001** |
| intensity_C * cover_C [1] | 0.00 | -0.00 – 0.00 | 0.13 | 0.900 |
| att_hijab_C * cover_C [1] | -0.00 | -0.00 – 0.00 | -0.02 | 0.982 |
| (emotion_C [1] * intensity_C) * att_hijab_C | -0.00 | -0.01 – 0.00 | -0.20 | 0.838 |
| (emotion_C [1] * intensity_C) * cover_C [1] | 0.00 | 0.00 – 0.01 | 2.66 | **0.008** |
| (emotion_C [1] * att_hijab_C) * cover_C [1] | -0.00 | -0.00 – 0.00 | -0.98 | 0.325 |
| (intensity_C * att_hijab_C) * cover_C [1] | -0.00 | -0.00 – 0.00 | -0.16 | 0.872 |
| (emotion_C [1] * intensity_C * att_hijab_C) * cover_C [1] | 0.00 | -0.00 – 0.00 | 0.40 | 0.690 |
| **Random Effects** | | | | |
| σ^2^ | 0.05 | | | |
| τ_00_ _subject_ | 0.02 | | | |
| τ_00_ _face_id_ | 0.00 | | | |
| τ_11_ _subject.emotion_C1_ | 0.00 | | | |
| τ_11_ _subject.intensity_C_ | 0.00 | | | |
| τ_11_ _subject.cover_C1_ | 0.00 | | | |
| τ_11subject.emotion_C1:intensity_C_ | 0.00 | | | |
| ρ_01_ _subject.emotion_C1_ | -0.01 | | | |
| ρ_01_ _subject.intensity_C_ | -0.17 | | | |
| ρ_01_ _subject.cover_C1_ | -0.03 | | | |
| ρ_01subject.emotion_C1:intensity_C_ | 0.21 | | | |
| ICC | 0.35 | | | |
| N _subject_ | 70 | | | |
| N _face_id_ | 8 | | | |
| Observations | 19337 | | | |
| Marginal R^2^ / Conditional R^2^ | 0.045 / 0.379 | | | |

1. The model on categorisation errors was chosen, as it had the highest overall fit (Conditional R^2^ of 0.83) compared to the models for the other DVs. [↑](#footnote-ref-1)
2. Choice ~ Emotion + Intensity + Attitude + Country + Emotion : Intensity + Emotion : Attitude + Emotion : Country + (Emotion + Intensity | subject) + (Emotion + intensity | Face) [↑](#footnote-ref-2)
3. Choice ~ Emotion * Intensity * Attitude * Country + (Emotion + Intensity | subject) + (Emotion + intensity | Face) [↑](#footnote-ref-3)
